# Inhaled Nitric Oxide Reverses Vascular and Respiratory Effects of ET-1 and PAF in Pigs

**DOI:** 10.1155/S0962935194000621

**Published:** 1994

**Authors:** Mariangela Albertini, Maria Giovanna Clement, Milena Dimori

**Affiliations:** Institute of Veterinary Physiology and Biochemistry University of Milan Via Celoria 10 Milan 20133 Italy

## Abstract

In anaesthetized paralysed, mechanically ventilated pigs, the vascular and respiratory effects of 80 ppm nitric oxide (NO) inhaled for 6 min were evaluated. To evoke different levels of smooth muscle contraction ET-1 or PAF, mediators involved in pulmonary disorders, were used. In control conditions, inhaled NO caused selective pulmonary vasodilatation without affecting respiratory resistances. This pulmonary vascular activity influenced the distensibility of the respiratory system and decreased inspiratory work. ET-1 administration significantly increased pulmonary arterial pressure and modestly changed mechanical properties of the respiratory system, while PAF caused potent vasoconstriction and bronchoconstriction associated with a marked change in volume-pressure relationship. In both cases, the changes in vascular and mechanical properties of the respiratory system increased inspiratory work. The vascular and respiratory activities of inhaled NO were correlated with preconstriction levels. The data show that the combination of vascular and respiratory effects improves pulmonary function, suggesting that inhalation of NO is a possible therapeutic approach for obstructive and inflammatory pulmonary diseases.


Mediators of Inflammation 3, 439–444 (1994)

### Inhaled nitric oxide reverses vascular and respiratory effects of ET-1 and PAF in pigs

Mariangela Albertini,[CA]
Maria Giovanna Clement and Milena Dimori

Institute of Veterinary Physiology and Biochemistry, University of Milan, Via Celoria 10, 20133 Milan, Italy

[CA] Corresponding Author

**In anaesthetized paralysed, mechanically ventilated pigs, the vascular and respiratory effects of 80 ppm nitric oxide (NO) inhaled for 6 min were evaluated. To evoke different levels of smooth muscle contraction ET-1 or PAF, mediators involved in pulmonary disorders, were used. In control conditions, inhaled NO caused selective pulmonary vasodilatation without affecting respiratory resistances. This pulmonary vascular activity influenced the distensibility of the respiratory system and decreased inspiratory work. ET-1 administration significantly increased pulmonary arterial pressure and modestly changed mechanical properties of the respiratory system, while PAF caused potent vasoconstriction and bronchoconstriction associated with a marked change in volume–pressure relationship. In both cases, the changes in vascular and mechanical properties of the respiratory system increased inspiratory work. The vascular and respiratory activities of inhaled NO were correlated with preconstriction levels. The data show that the combination of vascular and respiratory effects improves pulmonary function, suggesting that inhalation of NO is a possible therapeutic approach for obstructive and inflammatory pulmonary diseases.**

**Key words**: Endothelin-1, Nitric oxide, Platelet activating factor, Respiratory mechanics

### Introduction

The discovery of endothelium-derived relaxing factor and its subsequent identification as nitric oxide (NO) revealed a new regulatory mechanism for bronchomotor and vascular tone.[1–8] NO has been detected in exhaled air, with stable concentrations at rest and increased concentrations after challenges in sensitized animals with antigen or in asthmatic patients.[9] This implies that NO is endogenous and it may be one of the recently described epithelium-derived relaxing factors.[10,11]

Recently, it has been demonstrated that NO synthase can be induced by endogenous platelet activating factor (PAF).[12] This is a phospholipid mediator that causes bronchoconstriction, lung inflammation and hyperresponsiveness. PAF is released by inflammatory cells in obstructive, inflammatory and asthmatic disorders.[13] Therefore, it has been suggested that PAF might be a mediator of endothelin-induced bronchoconstriction in the guinea-pig.[14] Endothelin-1 (ET-1) is a potent vasoconstrictor[15] and bronchoconstrictor peptide[16–18] produced by endothelial cells and degraded predominantly in the pulmonary vasculature. ET-1, like PAF, also has a pathophysiological role. Immunoreactive ET-1 has been recovered in bronchoalveolar lavage fluid of asthmatic patients and high plasmatic levels of this peptide have been found in adult respiratory distress syndrome (ARDS).[19]

ET-1 activates the cyclooxygenase pathway[20] and induces release of endothelial-derived relaxing factor. Recently it has been suggested that inhalation of NO might be used as a therapeutic strategy to reduce symptoms of obstructive and hypertensive pulmonary disorders.[21,22] These last, changing the mechanical properties of the respiratory system, can increase the respiratory work, with a limiting effect on pulmonary activity.

The purpose of this study was to evaluate the effects of inhaled NO on mechanical properties of respiratory system during bronchoconstriction and pulmonary hypertension caused by administration of ET-1 or PAF, mediators involved in pulmonary disorders. We have also used ET-1 and PAF to see whether or not NO activity is correlated with the degree of bronchoconstriction.

### Materials and Methods

Twelve Large White pigs, of either sex, weighing 20.4 ± 1.7 (SEM) kg, were used. The animals, sedated with 1% propionylpromazine hydrochloride (0.05 ml/kg, i.m.) were anaesthetized with 15 mg/kg thiopental-sodium injected into the auricular vein. The depth of anaesthesia was maintained by infusion, drop by drop, of thiopental-sodium (9 mg/kg/h). The animals, tied in the supine position on a heated operating table, were tracheostomized, para-

© 1994 Rapid Communications of Oxford Ltd lysed with pancuronium bromide (0.02 mg/kg, i.v.) and mechanically ventilated (Servoventilator Siemens 900 C). When necessary, additional paralysing drug was administered during the experiments.

An endotracheal tube was inserted into the lower portion of the extrathoracic trachea. A heated pneumotachograph (Fleisch No 2, Fleisch Lausanne, Switzerland) was connected to the proximal end of the endotracheal tube and the 'Y' piece of the ventilator to evaluate respiratory flow. Electronic integration of the flow signal gave the tidal volume. The pressure drop across the two ports of the pneumotachograph was measured with a differential pressure transducer (Statham PM15, 10846). The response of the pneumotachograph was linear over the experimental range of flows. To reduce the effects of the compliance of the system on the mechanical measurements, a fixed length standard low-compliance tube was used (2 cm ID, 60 cm long) to connect the animals to the ventilator. The equipment flow resistance was 0.5 cm $H_2O$/l/s and the equipment dead space was 29.5 ml.

A balloon-tipped catheter (Swan Ganz 5F) was introduced into the left brachial vein and allowed to float through the right heart to the pulmonary artery. Polyethylene catheters were inserted into the right femoral artery to record blood pressure and into the right femoral vein for drug administration. Systemic and pulmonary arterial pressure were recorded by connecting the catheters to a fluid-filled capacitance manometer (Bell & Howell 4-422). All parameters were calibrated independently and simultaneously recorded on a multichannel pen recorder (model 8K40; NEC San-Ei Instruments, Ltd, Tokyo, Japan). Heart rate, mean arterial pressure and mean pulmonary pressure (MPAP) were evaluated from the polygraph tracings.

*Procedure and data analysis*: The baseline ventilator settings were a fixed inflation volume of 0.2 ± 0.01 (SEM) l and a fixed inspiratory flow of 0.25 ± 0.01 (SEM) l/s. Respiratory frequency was 23 ± 2 (SEM) breaths/min and the ratio of inspiratory time to total breathing cycle duration was 0.33 ± 0.01 (SEM). Respiratory mechanics values were assessed by the constant flow inspiratory occlusion method.[23] For each breath, airway occlusion was followed by a rapid initial drop in tracheal pressure and was maintained until an apparent plateau, representing the end-inspiratory elastic recoil pressure, was achieved (5–6 s). The difference between the peak of tracheal pressure and plateau pressure, divided by the immediately preceding steady flow, provided the total resistance of the airways (Rrs). The inspiratory work (W) of respiratory system was evaluated as area of the volume–pressure changes ($\Delta V/\Delta P$).

*Protocol*:

1. After evaluation of control values, obtained when the animals were breathing air through the servoventilator, nitric oxide (NO) was administered through the servoventilator for 6 min. The inspired gas was a precise mixture of oxygen and nitrogen immediately diluted with NO to produce the desired concentration of inspired NO (80 ppm). With volumetrically calibrated flowmeters, NO in the bag (mixture of 235 ppm NO in pure $N_2$) was substituted for pure $N_2$ to give the desired concentration of inspired NO at a concentration of inspired oxygen ($FIO_2$) of 0.6–0.7. We used 60–70% oxygen to avoid hypoxic vasoconstriction.
2. After recovery to baseline values, six of the 12 pigs were treated with ET-1, administered through the Swan-Ganz catheter at a dose of 200 pmol/kg, as previously reported.[24] NO was inhaled at the peak of ET-1 dependent vascular and respiratory effects (about 10 min).
3. After recovery to baseline values, the other six pigs were treated with PAF, freshly mixed in normal saline solution, administered i.v. at a dose of 50 ng/kg. This dose, as previously reported,[25] was sufficient to ensure significant and reproducible haemodynamic and respiratory effects in pigs. The activity of inhaled NO was evaluated at the peak of PAF-dependent vascular effects (5 min).

*Data analysis and statistics*: Results are expressed as means ± SEM. The significance of differences between two sets of data was assessed by the two-tailed *t*-test for paired data. A significant difference was defined as $P < 0.05$.

### Results

In all experimental conditions inhaled NO did not modify systemic blood pressure or heart rate, but changed pulmonary vascular pressure and mechanical properties of respiratory system.

Figure 1A shows that in control conditions NO inhalation caused a significant decrease in pulmonary vascular pressure. This decrease in MPAP was associated with a change in the $\Delta V/\Delta P$ relationship of the respiratory system, as evidenced in Fig. 1B. In control conditions this volume–pressure relationship, although linear between 0.05 and 0.2 l, exhibited a 'knee' at the lower volumes. Inhaled NO modified the slope (from 19.07 to 20.17 l/cm $H_2O$) but did not affect the 'knee' or the linearity of the curve. The change in slope after NO inhalation reflects improvement of the pulmonary function, that is, significantly decreases respiratory system work (Fig. 1C), while respiratory resistance did not change (Fig. 1D).

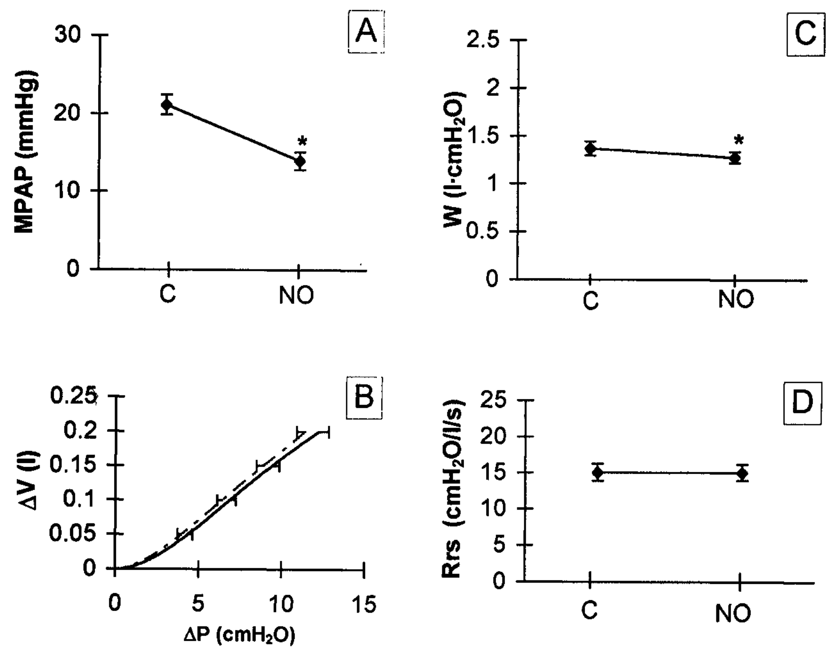

FIG. 1. Effects of inhaled NO in control conditions (C). (A) Mean pulmonary arterial pressure (MPAP); (B) average volume–pressure (ΔV/ΔP) relationships of respiratory system obtained in control conditions (—) and after NO inhalation (----) evaluated at fixed volumes; above 0.05 l, lines were computed by linear regression analysis. (C) Inspiratory work of respiratory system (W); (D) respiratory resistance (Rrs). Values are expressed as means ± SEM. *$p < 0.05$ NO vs. control conditions.

*Effects of NO inhalation after ET-1 administration*: NO inhaled after ET-1 administration completely counteracted pulmonary hypertension and the changes in mechanical properties of respiratory system evoked by ET-1.

The changes in mean pulmonary arterial pressure due to ET-1 administration and NO inhalation are reported in Fig. 2A. The data show that the administration of ET-1 significantly increased MPAP (141.32 ± 2.96%), which was completely counteracted by NO inhalation, which brought the MPAP to values lower than controls (78.6 ± 6.37%).

Figure 2B shows the ΔV/ΔP relationship obtained in control conditions, after treatment with ET-1 and after NO inhalation. The modest displacement of this relationship caused by ET-1 administration was completely reversed by inhalation of nitric oxide.

The changes in respiratory system work are reported in Fig. 2C. The increased respiratory work (107.13 ± 2.41%) caused by ET-1 administration was completely restored to control values (98.76 ± 2.93%) by NO inhalation. Changes in respiratory work were associated with changes in the respiratory resistances (Fig. 2D). In fact, ET-1 administration modestly increased Rrs. Even though this change was not significant, inhaled NO reduced Rrs toward control values.

*Effects of NO inhalation after PAF administration*: PAF caused marked pulmonary vasoconstriction and changes in the mechanical properties of respiratory system. NO inhaled after PAF exerted a strong relaxing effect, and, though only partially, counteracted the pulmonary vascular and respiratory effects due to PAF administration. The significant PAF-dependent increase in pulmonary arterial pressure (274.3 ± 15.22%) was almost completely restored to control values (122.6 ± 5.96%) by NO inhalation (Fig. 3A).

The slope and the linearity of the ΔV/ΔP curve observed in control conditions were greatly affected by PAF administration, as evidenced in Fig. 3B. In this case too, inhalation of nitric oxide reduced the PAF-dependent changes, without restoring the linearity of the curve.

The changes in respiratory system work caused by PAF administration and NO inhalation are reported in Fig. 3C. Data show that the PAF-dependent significant increase in work (171.6 ± 10.7%) was significantly reduced by NO inhalation (118.1 ± 4.56%), without restoring the respiratory work to the control values. The resistance of the respiratory system after PAF administration and NO inhalation is reported in Fig. 3D. The increase in Rrs of 320.88 ± 26.05% due to PAF was partially counterbalanced by NO inhalation, which restored Rrs near to the control values (145.97 ± 11.14%).

### Discussion

This study demonstrates that in anaesthetized, paralysed pigs inhaled NO acts as a selective pulmonary vasodilator and improves the mechanical properties of the respiratory system. These effects are present in control conditions and also after pretreatment with ET-1 or PAF, two agents involved

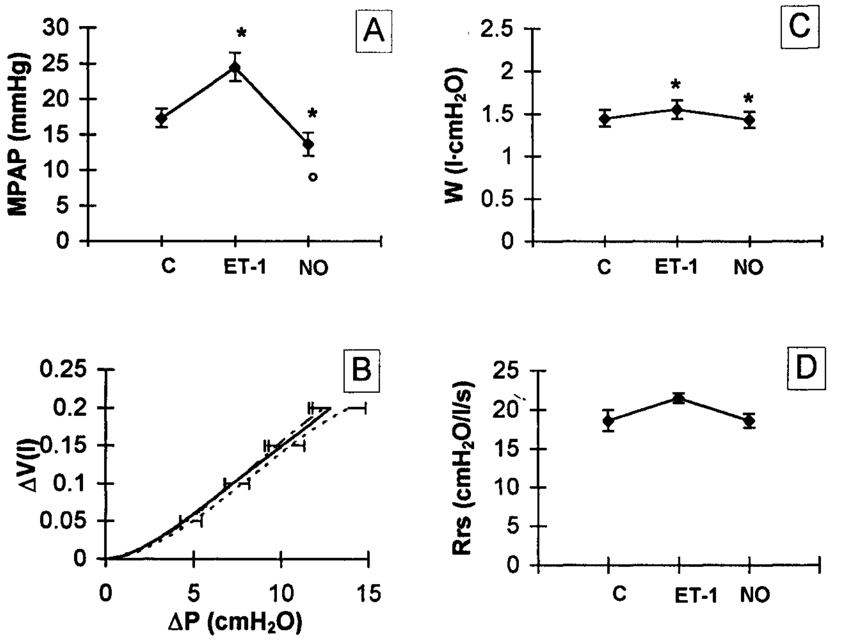

FIG. 2. Effects of NO inhaled after ET-1 administration. (A) Mean pulmonary arterial pressure (MPAP); (B) volume–pressure (ΔV/ΔP) relationships of respiratory system obtained in control conditions (—), after ET-1 administration (----) and after NO inhalation (-—-). (C) Inspiratory work of respiratory system (W); (D) respiratory resistance (Rrs). Values are expressed as means ± SEM. *$p < 0.05$ ET-1 or NO vs. control conditions. °$p < 0.05$ NO vs. ET-1 administration.

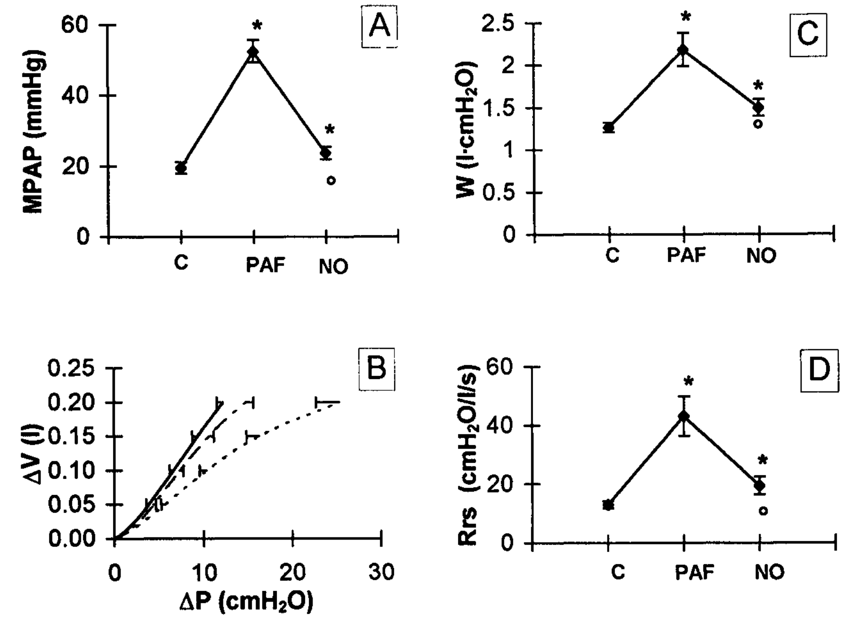

FIG. 3. Effects of NO inhaled after PAF administration. (A) Mean pulmonary arterial pressure (MPAP); (B) volume–pressure (ΔV/ΔP) relationships of respiratory system obtained in control conditions (—), after PAF administration (----) and after NO inhalation (-—-). (C) Inspiratory work of respiratory system (W); (D) respiratory resistance (Rrs). Values are expressed as means ± SEM. *$p < 0.05$ PAF or NO vs. control conditions. °$p < 0.05$ NO vs. PAF administration.

in many respiratory diseases characterized by bronchoconstriction and pulmonary hypertension.

ET-1, known as a potent vasoconstrictor and bronchoconstrictor agent,[15–20] in the pig causes only modest contraction of airways smooth muscles but pulmonary hypertension similar to that observed in other species.[24,26,27] As suggested by Battistini *et al.*,[14] the bronchopulmonary action of ET-1 may be mediated by endogenous release of PAF which, like endothelin, has a pathophysiological role in lung dysfunctions. In the pig, 50 ng/kg PAF is a strong constrictor of pulmonary vascular and bronchial smooth muscles.[25]

An important new regulatory mechanism of the pulmonary and systemic circulation[5–8] is attributed to nitric oxide, also known as endothelium-derived relaxing factor. Recent observations demonstrate that when NO is inhaled, it acts as a selective local pulmonary vasodilator, without causing systemic vasodilatation.[6,7,28]

Our results confirm these findings and also suggest that in the pig NO has selective pulmonary vasodilator activity not only on preconstricted vascular smooth muscle, but also on normal vascular tone. Using ET-1 and PAF, which cause two different degrees of contraction, we also demonstrated that the NO vasodilator activity is correlated with the degree of vasoconstriction, because its effect is greater after administration of PAF, which causes a stronger pulmonary hypertension. The NO-dependent pulmonary vasodilator activity improves respiratory function. In fact, in control conditions the vasodilator activity of inhaled NO improves the distensibility of the respiratory system, as evidenced by displacement of the $\Delta V/\Delta P$ relationship, even if it did not affect respiratory resistance. The 'knee' observed in the $\Delta V/\Delta P$ curve at lower volumes in anaesthetized, paralysed, mechanically ventilated pigs, similarly to that observed in cats,[29] reflects different stresses within the tissue elements. The persistence of this 'knee' when NO is inhaled in control conditions shows that stress inequalities are not affected by NO, which essentially acts on vascular smooth muscle.

The area enclosed between the $\Delta P$ vs. $\Delta V$ curve represents the inspiratory work of the respiratory system.[30] The displacement of the $\Delta V/\Delta P$ curve to the left by NO is an index of decreased work done by the respiratory system. Therefore, the present results show that when 80 ppm NO is inhaled for 6 min, it improves pulmonary vascular and mechanical functions of the respiratory system. The vasodilatation associated with the improvement of lung distensibility and with the decrease in inspiratory work favours the redistribution of blood flow to better ventilated areas of the lungs, improving gas exchange.[31,32] A marked improvement of oxygen exchange and arterial oxygen tension caused by inhalation of 5–80 ppm of NO was also seen recently by Falke *et al.*[33] in patients with ARDS and by Roberts *et al.*[34] in neonates with persistent pulmonary hypertension of the newborn. Recent observations have shown that inhaled NO causes bronchodilatation and not simply vasodilatation of ventilated lung regions.[21] In fact, inhaled NO molecules are extremely lipophilic[35] and can diffuse through the bronchial epithelial barrier to reach airway smooth muscle and to produce airway relaxation.

In the pig, bronchodilatation appears only when airways smooth muscles are precontracted, because changes in Rrs were observed only when NO was inhaled after ET-1 or PAF administration. The bronchial preconstriction level influences the degree of response to inhaled NO similarly to that of the pulmonary vascular bed. When NO was inhaled after ET-1, causing modest contraction, the bronchomotor and the vascular tones were completely restored. When NO was inhaled after PAF administration, exerting more potent vasoconstriction and bronchoconstriction, the vasodilator and bronchodilator effects were greater, even though NO only partially reversed PAF-dependent action.

The complex vasoactive and respiratory effects of PAF are characterized not only by pulmonary hypertension and potent bronchoconstriction, but also by lung oedema[36,37] and decreased lung distensibility.[38] Oedema and parenchymal damage due to PAF administration may alter the linearity of the $\Delta V/\Delta P$ relationship, also in the range between 0.05–0.2 l. The displacement to the right and the non-linearity of the $\Delta V/\Delta P$ curve observed after PAF evidence the reduction in pulmonary compliance and the non-homogeneous lung distensibility. Inhaled NO, by its vasodilator activity, reduces pulmonary PAF-dependent hypertension and decreases the accumulation of extravascular lung water. This effect, associated with marked bronchodilator activity, is responsible for the great displacement of the $\Delta V/\Delta P$ curve to the left. Although inhaled NO does not completely restore the slope and the linearity of the curve and only partially reduces the PAF-dependent bronchoconstriction, the changes observed in the mechanical properties of the respiratory system suggest that inhaled NO might be a potent therapeutic approach for hypertensive and obstructive lung diseases.

It is known that obstructive pulmonary diseases impose significant amounts of respiratory work. During a breathing cycle, work must be done to overcome flow-resistive forces, the magnitude of which depends on the flow-resistance and respiratory frequency.[39] To avoid the frequency-dependence of work, we ventilated the pigs at constant flow and volume, and to induce two different degrees of bronchoconstriction, causing different flow resistances, we used ET-1 and PAF. A greater flow-resistance requires a greater pressure-gradient and hence more work to overcome flow-resistive forces. PAF causing an increase in respiratory resistance more marked than ET-1 does, induces a greater increase in inspiratory work. Therefore, NO's vasodilator activity, associated with a marked decrease in respiratory resistance and improved lung distensibility, favours pulmonary function, reducing inspiratory work. In this case, too, NO activity is correlated with the degree of mechanical impairment of the respiratory system, as evidenced by the greater decrease in inspiratory work observed when NO is inhaled after PAF.

In conclusion, the present data gives evidence that nitric oxide causes not only selective pulmonary vasodilatation but also bronchodilatation. Pulmonary vasodilatation favours arterial oxygenation and reduction of pulmonary oedema, while bronchodilatation, due to a direct action of nitric oxide on airways smooth muscle, reduces respiratory resistance. The only partial reduction of vascular and respiratory PAF-dependent changes suggests that the response to inhaled NO is dose- and time-dependent. The association of vascular and bronchial effects improves lung distensibility, reducing respiratory work. These advantages, associated with rapidity of effects and ease of administration, in the absence of systemic hypotensive effects, make inhaled NO a therapeutically important approach, not only in obstructive respiratory distress syndrome but also in inflammatory allergic endothelin-1 and/or platelet activating factor-mediated diseases.

### References

1. Moncada S, Palmer RMJ, Higgs EA. Nitric oxide: physiology, pathophysiology and pharmacology. *Pharmacol Rev* 1991; **43:** 108–142.
2. Barnes PJ. Nitric oxide and airways. *Eur Resp J* 1993; **6:** 163–165.
3. Belvisi MG, Stretton CD, Yacoub M, Barnes PJ. Nitric oxide is the endogenous neurotransmitter of bronchodilator nerves in humans. *Eur J Pharmacol* 1992; **210:** 221–222.
4. Tucker JF, Brane SR, Charalambons L, Hobbs AJ, Gibson A. L-$N^G$-Nitro arginine inhibits non-adrenergic, non cholinergic relaxation of guinea pig isolated tracheal smooth muscle. *Br J Pharmaol* 1990; **100:** 663.
5. Frostell CG, Blomqvist H, Lundberg J, Hedenstierna G, Zapol WM. Inhaled nitric oxide dilates human hypoxic pulmonary vasoconstriction without causing systemic vasodilation. *Anesthesiology* 1991; **75** (suppl): A989.
6. Frostell C, Fratacci MD, Wain JC, Jones R, Zapol WM. Inhaled nitric oxide—a selective pulmonary vasodilator reversing hypoxic pulmonary vasoconstriction. *Circulation* 1991; **83:** 2038–2047.
7. Fratacci MD, Frostell C, Chen TY, Wain JC Jr, Robinson DR, Zapol WM. Inhaled nitric oxide—a selective pulmonary vasodilator of heparin-protamin vasoconstriction in sheep. *Anesth (Laboratory Investigations)* 1991; **75:** 990–999.
8. Ryan US, Rubanyi GM, eds. *Endothelial Regulation of Vascular Tone.* New York: Marcel Dekker, 1992.
9. Kharatinov SA, Yates D, Robbins RA, Logan-Sinclair R, Shinebourne EA, Barnes PJ. Increased nitric oxide in exhaled air of asthmatic patients. *Lancet* 1994; **343:** 133–135.
10. Barnes PJ. Invited editorial on 'Epithelium acts as a modulator and a diffusion barrier in the responses of canine airway smooth muscle'. *J Appl Physiol* 1994; **76:** 1841–1842.
11. Munakata M, Masdaki Y, Ukita H, Homma Y, Kawakami Y. Is epithelium-derived relaxing factor (EpDRF) also nitric oxide (NO)? *Am Rev Resp Dis* 1989; **139** (suppl): A351.
12. Szabó C, Wu C-C, Mitchell JA, Gross SS, Thiemermann C, Vane JR. Platelet-activating factor contributes to the induction of nitric oxide synthase by bacterial lipopolysaccharide. *Circ Res* 1993; **73:** 991–999.
13. Braquet P, Touqui L, Shen TY, Vargaftig BB. Perspectives in platelet-activating factor research. *Pharmacol Rev* 1987; **39:** 97–145.
14. Battistini B, Sirois P, Braquet P, Filep JG. Endothelin-induced constriction of guinea pig airways: role of platelet-activating factor. *Eur J Pharmacol* 1990; **186:** 307–310.
15. Rubanyi GM, Cedar Knolls NJ, Vanhoutte PM, eds. *Endothelium-derived Contracting Factors.* Basel: Karger, 1990.
16. Uchida Y, Ninomiya H, Saotome M. *et al.* Endothelin a novel vasoconstrictor peptide as a potent bronchoconstrictor. *Eur J Pharmacol* 1988; **154:** 227–228.
17. Nomura A, Uchida Y, Ohtsuka M, *et al.* Endothelium-derived polypeptide potently constricts human bronchi. *Am Rev Respir Dis* 1989; **139:** A468.
18. Grunstein MM, Chuang ST, Schramm CM, Pawlowski NA. Role of endothelin 1 in regulating rabbit airway contractility. *Am J Physiol* 1991; **260:** L75–L82.
19. Druml W, Steltzer H, Waldhäusl W, *et al.* Endothelin-1 in adult respiratory distress syndrome. *Am Rev Respir Dis* 1993; **148:** 1169–1173.
20. Horgan M, Pinheiro JMB, Malik AB. Mechanism of endothelin-1-induced pulmonary vasoconstriction. *Circ Res* 1991; **69:** 157–164.
21. Dupuy PM, Shore SA, Drazen JM, Frostell C, Hill WA, Zapol WM. Bronchodilator action of inhaled nitric oxide in guinea pigs. *J Clin Invest* 1992; **90:** 421–428.
22. Rossaint R, Falke KJ, López F, Slama K, Pison U, Zapol WM. Inhaled nitric oxide for the adult respiratory distress syndrome. *N Engl J Med* 1993; **328:** 399–405.
23. Bates JHT, Rossi A, Milic-Emili J. Analysis of the behaviour of the respiratory system with constant inspiratory flow. *J Appl Physiol* 1985; **58:** 1840–1848.
24. Clement MG, Dimori M. Inhaled nitric oxide (NO) counterbalances ET-1-dependent pulmonary hypertension and bronchoconstriction in the pig. *Mediators Inflamm* 1994; **3:** 131–135.
25. Aguggini G, Berti F, Clement MG, Magni F, Rossoni G. The ginkgolide BN 52021 antagonizes the increase in pulmonary pressure induced by platelet activating factor in pig: a link to $TXA_2$ generation. In: Braquet P, ed. *Ginkgolides—Chemistry, Biology, Pharmacology and Clinical Perspective, Vol 2.* Barcelona: Prous J R Science Publishers, 1989.
26. Clement MG, Dimori M, Albertini M. Influence of the plasma ET-1 level on the vascular and respiratory systems of the pig. *Biomed Res (India)* 1994; **5:** 19–27.
27. Clement MG, Dimori M, Albertini M. Comparison of vascular and respiratory effects of endothelin-1 in the pig. *Mediators Inflamm* 1993; **2:** 287–292.
28. Pepke-Zaba J, Higenbottam TW, Dinh-Xuan AT, Stone D, Wallwork J. Inhaled nitric oxide as a cause of selective pulmonary vasodilation in pulmonary hypertension. *Lancet* 1991; **338:** 1173–1174.
29. Kochi T, Okubo S, Zin WA, Milic-Emili J. Chest wall and respiratory system mechanics in cats: effects of flow and volume. *J Appl Physiol* 1988; **64:** 2636–2646.
30. D'Angelo E, Calderini E, Torri G, Robatto FM, Bono D, Milic-Emili J. Respiratory mechanics in anesthetized paralyzed humans: effects of flow, volume, and time. *J Appl Physiol* 1989; **67:** 2556–2564.
31. Pison U, Lopez FA, Heidelmeyer CF, Rossaint R, Falke KJ. Inhaled nitric oxide reverses hypoxic pulmonary vasoconstriction without impairing gas exchange. *J Appl Physiol* 1993; **74:** 1287–1292.
32. Rovira I, Chen T-Y, Winkler M, Kawai N, Bloch KD, Zapol WM. Effects of inhaled nitric oxide on pulmonary hemodynamics and gas exchange in an ovine model of ARDS. *J Appl Physiol* 1994; **76:** 345–355.
33. Falke K, Rossaint R, Pison U, *et al.* Inhaled nitric oxide selectively reduces pulmonary hypertension in severe ARDS and improves gas exchange as well as right heart ejection fraction: a case report. *Am Rev Respir Dis* 1991; **143:** 248a (abstract).
34. Roberts JD, Polaner DM, Todres ID, Lang P, Zapol WM. Inhaled nitrix oxide: a selective pulmonary vasodilator for the treatment of persistent pulmonary hypertension of the newborn. *Circulation* 1991; **84** (suppl II): 1279a (abstract).
35. Young CL. Solubility data. Nitrix oxide-organic liquids system. *Solubility Data Series* 1981; **8:** 336–351.
36. Filep JG, Földes-Filep É. Modulation by nitric oxide of platelet-activating factor-induced albumin extravasation in the conscious rat. *Br J Pharmacol* 1993; **110:** 1347–1352.
37. Chen C-R, Voelkel NF, Chang SW. Pulmonary vascular reactivity: effect of PAF and PAF antagonists. *J Appl Physiol* 1992; **73:** 1762–1769.
38. Clement MG, Albertini M, Dimori M, Aguggini G. PAF and the role of the vagus nerve in the breathing pattern of the pig. *Prost Leuk Ess Fatty Acid* 1992; **45:** 143–149.
39. Otis AB. The work of breathing. In: Fenn WO, Rahn H, eds. *Handbook of Physiology.* Washington: American Physiological Society. 1964, 463–476.

**Received 5 July 1994; accepted 4 August 1994**
